# Association of suicidal ideation and depression with the use of proton pump inhibitors in adults: a cross-sectional study

**DOI:** 10.1038/s41598-022-24244-z

**Published:** 2022-11-14

**Authors:** Pedro Fong, Sut Tong Chan, Pui Nap Lei, Hao Ian Cheong, I Man Cheong, Weng Lam Hoe

**Affiliations:** School of Health Sciences and Sports, Macao Polytechnic University, Meng Tak Building, Room 705, Rua de Luís Gonzaga Gomes, Macao, China

**Keywords:** Epidemiology, Drug safety

## Abstract

Proton pump inhibitors (PPIs) were found to be associated with depression. This study aimed to find the cross-sectional association between recent PPI use and suicidal ideation. Item 9 of Patient Health Questionnaire-9 (PHQ-9) of the US National Health and Nutrition Examination Survey (NHANES) between 2005 and 2018 was used to categorize whether or not the participants had suicidal ideation. The secondary outcome of this study was depression and the scores of the PHQ-9 were used as the depression diagnostic instrument. The study population included 16,881 participants who were over 20 years old. The bivariate Rao-Scott χ^2^ test showed a significant association between PPI use and suicidal ideation (*P* < 0.001) and a stronger association was observed between PPIs and depression (*P* < 0.001). Multiple logistic regression analysis of the education, gender, race and age-adjusted model revealed that the PPI users had a 2.34 (95% CI 1.66–3.31) greater risk of having suicidal ideation than the non-PPI users. Middle-aged participants (40–49 years) showed the greatest number of differences in suicidal ideation between PPI and non-PPI users (*P* < 0.001). Future research should continue to consider the psychiatric effects of taking PPIs.

## Introduction

Proton pump inhibitors (PPIs) are one of the most successful therapeutic agents to treat gastric acid-related disease^1^. They are also one of the most frequently prescribed drugs for gastric ulcers and can be purchased over-the-counter without a prescription in many countries^2^. However, studies have shown that 40–60% of PPI use is inappropriate, mainly misused or overused^3^. PPI has been inappropriately used to treat unlicensed indications or use for an unnecessarily prolonged period. For example, PPI should not be used for long-term gastric ulcer prophylaxis in patients without risk factors and prolonged use for functional dyspepsia^3^. Although PPIs have demonstrated an adequate safety profile through many years of clinical use, studies have found that there are some potential risks with long-term use. PPIs may increase the risk of infections and secondary hypergastrinemia, along with reduced absorption of micronutrients such as calcium, iron and vitamin B_12_^4,5^. Thus, guidance has been published for prescribers in order to prevent the inappropriate use of PPIs^6,7^.

PPIs were also found to have neuropsychological effects, however the findings between different studies had some degree of contradiction. A large prospective cohort study on 73,679 participants who were aged 75 years or older concluded that PPI use can significantly increase the risk of dementia (hazard ratio: 1.44; 95% CI 1.36–1.52)^8^. In contrast, a large meta-analysis conducted on six selected cohort studies with participants who were aged 55 years or older suggested that there was no association between dementia and PPI use^9^. A recent study with 1573 participants suggested that PPIs can affect cognitive performance diversely in different people depending on their genes^10^. The study assessed the participants’ medical histories, blood samples and neuropsychological conditions. Participants with the APOE4 allele were found to have increased performance in certain types of cognitive functions after taking PPIs, whereas these performances declined in non-APOE4 carriers after PPI use.

Studies have also indicated an association between PPIs and depression^11,12^. Laudisio et al*.* assessed the mood of 344 participants who were over 75 years old and taking PPIs using the Geriatric Depression Scale^12^. Their study indicated a significant association between PPIs and depression (OR: 2.38; 95% CI 1.02–5.58) and this association was independent of the type of gastric disease. The pharmacological mechanisms of these neuropsychological effects of PPIs are still unknown but they may be linked to the reduction of vitamin B_12_ absorption. Vitamin B_12_ is an important element for maintaining the features of neurological systems and a deficiency may cause cognitive impairment, dementia or even Alzheimer’s disease^13^. One study investigated the relationship between vitamin B_12_ deficiency and depression in 700 elderly participants using the Geriatric Depression Scale and the results concluded that participants with B_12_ deficiency were 2.05 times more likely to have severe depression^14^.

Suicidal ideation is generally described as thoughts or self-absorption toward death. The clinical characteristics, severity and duration of SI may vary remarkably on the individual at different episodes^15^, ranging from a brief desire of never wake up in the morning to an intensive impulse of self-annihilation. Suicidal ideation may recognise as a heterogeneous phenomenon with a fluctuating pattern, and healthcare professionals should assess and monitor the severity and nature of this pattern^15^. The rate of attempting suicide in an individual with suicidal ideation is low, about 3.5% in Americans^15^. This rate affects by many factors, including age, race, education level and psychological condition^16^. Suicidal ideation can sometimes be considered a symptom of depression. An association between suicidal ideation and depression has been shown in one study, with people having both suicidal ideation and depression being more likely to attempt suicide^16^.

The pathophysiology of depression involves multiple interactions between the central nervous system and the immune system^17^. Factors that activate the immune system may cause changes in the interactions between the hypothalamus, pituitary gland, and adrenal gland, which may increase the secretion of glucocorticoids and inflammatory cytokines, affecting the brain monoamine, glutamate and neuropeptide systems. This may eventually lead to depression or other neuropsychiatric disorders^18,19^. An example is that air pollutant triggers an immune response in an asthmatic patient may increase the release of inflammatory cytokines and initiate changes in the hypothalamic–pituitary–adrenal (HPA) axis and produce psychological effects, such as depression^20,21^.

According to the authors, no studies have been performed on a national dataset to find the association between PPIs and suicidal ideation or depression. This study aimed to investigate the association of PPIs on depression and suicidal ideation using the US National Health and Nutrition Examination Survey (NHANES).

## Methods

### Data sources

All data used in this study were obtained from the NHANES dataset, which was conducted by the National Center for Health Statistics (NCHS) of the Centers for Disease Control and Prevention (CDC). NHANES is a continuous 2-year cyclic cross-sectional programme aimed at measuring the well-being of non-institutionalized US adults and children. The data of this study were obtained from seven cycles between 2005 and 2018. The participants of the survey were selected using complex, stratified and multistage sampling methods^22–25^. Sample weights were carefully adjusted for each participant to minimize oversampling and to ensure the survey can adequately represent the US nation. All participants gave informed consent for taking part in the survey and the data collection processes were approved by the NCHS Ethics Review Board.

**Figure 1:**
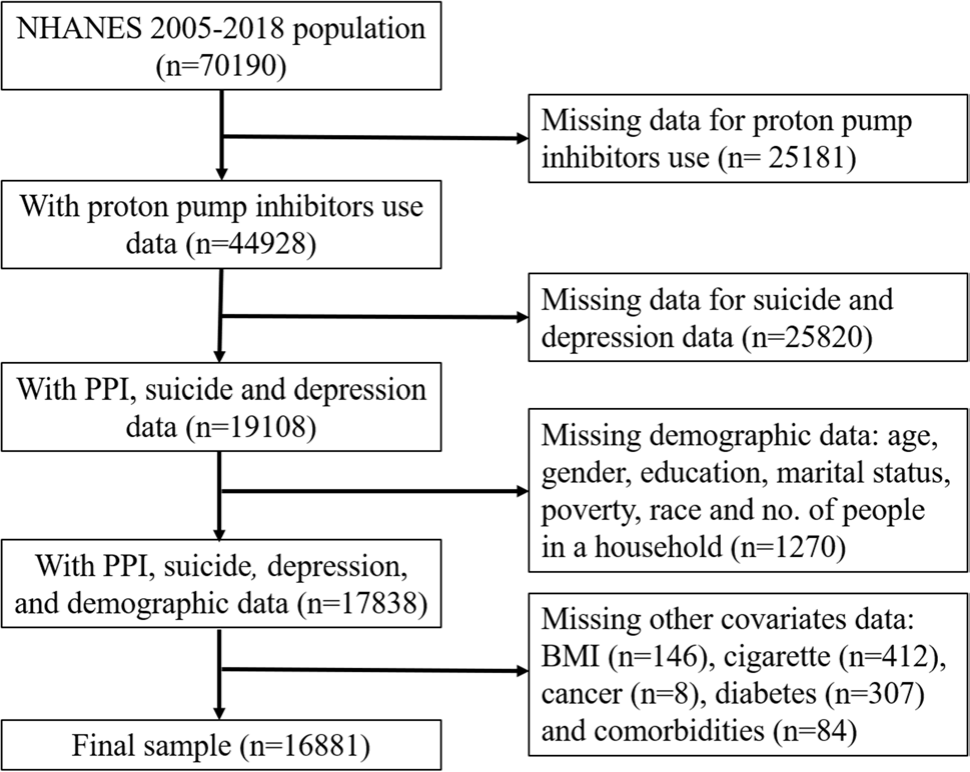
Flow chart of the study populations.

Most NHANES data are publicly available and categorized into five sections: demographic, dietary, examination, laboratory and questionnaire. Each of these sections has different subcategories. The data for this study were obtained from the questionnaire section, in which the suicidal ideation and depression data were obtained from the ‘Mental Health – Depression Screener’ subcategory^26^ and the PPI data from the ‘Prescription Medications’ subcategory^27^. The number of participants with demographic data in the NHANES 2005–2018 was 70,109, of which 3806 had been taking PPIs in the past 30 days or more and 41,289 were not taken any medication. This study was aimed at adults from 20 years of age. After excluding all the missing data for all the variables, the final sample size was 16,881 (Fig. 1).

### Assessment of proton pump inhibitors

The exposure variable of this study is whether the participants had taken PPIs. Three NHANES interview questions were employed for obtaining this information: ‘*In the past 30 days, have you used or taken medication for which a prescription is needed?*’; ‘*Generic drug name?*’; and ‘*For how long have you been using or taking the drug?*’. The participants who had been taking PPIs for at least 30 days were selected for this study to ensure that the PPIs had enough exposure time to produce effects on the participants. From the data of the seven NHANES cycles (2005–2018), six PPIs were found: omeprazole (*N* = 1479), esomeprazole (*N* = 601), lansoprazole (*N* = 286), rabeprazole (*N* = 91), pantoprazole (*N* = 452) and dexlansoprazole (*N* = 51). The total number of participants with PPI use in the final sample was 2960. These participants may also have taken other medications besides PPI.

### Assessment of depression

The two outcome variables for this study were suicidal ideation and depression. Depression was defined by the score from the Patient Health Questionnaire-9 (PHQ-9)^28^, which is a validated self-reported diagnostic instrument for depression screening. It contains nine questions related to DSM-5 (Diagnostic and Statistical Manual of Mental Disorders)^29^ signs and symptoms. Each question is scored from 0 to 3, where 0 indicates no particular symptom in the past 2 weeks, 1 indicates symptoms on several days, 2 refers to symptoms on more than half the days and 3 refers to symptoms nearly every day. The addition of the scores from the nine questions has been used for assessing the severity of depression^30^. A total score of < 10 indicates no or mild depression whereas ≥ 10 indicates moderate or severe depression. For this study, the participants who obtained a score of ≥ 10 on the PHQ-9 self-report questionnaire were considered of having depression, whereas a score of < 10 indicated the participants had no depression.

The associations between PPI use and suicidal ideation were further analysed by stratifying participants under different depression severity groups. The total PHQ-9 scores of 5, 10, 15, and 20 were the cutpoints for the none-minimal, mild, moderate, moderately severe and severe depression groups, respectively^28^. Considering the depression severity as a categorical variable rather than a continuous variable enabled rational interpretations related to the levels of severity. The categorical variable also allowed a direct comparison of suicide ideation rates between PPI and non-PPI users under different severity levels.

### Assessment of suicidal ideation

The suicidal ideation outcome was defined by the ninth question of the PHQ: ‘*Over the last 2 weeks, how often have you been bothered by the following problems: Thoughts that you would be better off dead or of hurting yourself in some way?*’. This question has been considered as a strong predictor of suicidal behavior^31^. A score of 1, 2 or 3 indicated that the participants had suicidal ideation on several days, more than half of the days and nearly every day, respectively; 0 referred to no such thoughts. For this study, the participants who scored ≥ 1 were considered of having suicidal ideation, whereas a score of 0 indicated no suicidal ideation. The total number of participants with suicide ideation and depression data was 36,259. After combining these data with the PPI data, the sample size reduced to 19,108 (Fig. 1).

### Assessment of covariates

Covariates were selected based on the significant associations with suicidal ideation or depression found in the literature^32–34^. Demographic data such as age, race and gender were included. Personal data included body mass index (BMI), education level, marital status, number of people in the house, poverty income ratio (PIR) and smoking. Four BMI levels were assigned based on the CDC reference values^35^: < 18.5 kg/m^2^, underweight; 18.5–24.9 kg/m^2^, normal; 25.0–29.9 kg/m^2^, overweight; and > 30.0 kg/m^2^, obese. Participants were assigned as current smoker, previous smoker or never smoked. Previous smokers were those who were not currently smoking but had smoked at least 100 cigarettes in their life. With regard to the PIR, more than 5% of participants had missing PIR data and excluding them can substantially reduce the final sample size. The PIR was categorized into four groups: missing data, < 2, 2–4 and > 4. For all other covariates, missing data were excluded from the analysis. Health condition data included the number of comorbidities and history of cancer. The number of comorbidities was the sum of each of the diseases that the participants had: angina, coronary heart disease, heart failure, myocardial infarction and stroke. These diseases and also cancer were found to be associated with depression^36,37^. After combining all the available data and excluding the missing data, the final sample size of this study was 16,881 (Fig. 1), in which, the number of participants who had depression and suicidal ideation were 1158 and 510, respectively.

### Statistical analysis

R Studio (R version 4.1.0) was used to analyse the data. The survey sample weight was used according to the guidance^38^ of NHANES to account for survey non-response, oversampling and post-stratification adjustment, thus allowing the data to represent the US civilian non-institutionalized population adequately. The bivariate associations between suicide ideation or depression and all other categorical variables were evaluated using the Rao-Scott χ^2^ test^39^. The ‘survey’ package (https://cran.r-project.org/web/packages/survey/survey.pdf) of R was used to conduct multiple regression analysis and the ‘questionr’ package (https://cran.r-project.org/web/packages/questionr/questionr.pdf) was used to calculate the odds ratio (OR) and 95% confidence interval (CI). The covariates were adapted to develop logistic regression models by forwarding stepwise modelling. The covariates were introduced to the unadjusted model one at each time, starting from the demographic data (age, race and gender) and then personal information data (BMI, education level, marital status, number of people in house, PIR and smoking) and health condition data (number of comorbidities and history of cancer). The covariate stayed in the model if it improved the statistical significance and OR. This method assisted in fine-tuning the model by selecting the covariates.

### Ethics approval

All the data of this study were collected from the US National Health and Nutrition Examination Study (NHANES), which was ethically approved by the US National Center for Health Statistics (NCHS) Research Ethics Review Board (Protocol No. 2005-06, Continuation of Protocol No. 2005-06, Protocol No. 2011-17, Continuation of Protocol No. 2011-17, Protocol No. 2018-01). All methods were carried out in accordance with the principles of the Declaration of Helsinki. Informed, written consent was obtained from all participants.

## Results

The weighted sample distribution of the covariates stratified by suicide ideation or depression is presented in Table 1. Most of the samples were in the 20–29 (25.9%) and 30–39-year (22.7%) age groups and the majority were: non-Hispanic white (61.4%), married (52.7%), never smoked (56.6%), without cancer (91.2%), non-diabetic (95.7%) and no comorbidities (95.6%). Several groups of the covariates had a minor weighted sample, for instance, 1.7% were underweight, 1% had two comorbidities and 0.9% had three or more comorbidities.

**Table 1:** Weighted characteristics of 16,881 participants by suicidal ideation and depression, NHANES 2005–2018.

Variable	Weighted participants (%)	Suicide ideation		*p*-values^a^	Depression		*p*-values^a^
		Yes (%)	No (%)		Yes (%)	No (%)	
All	100	2.7	97.3		6.3	93.7	
**Protein pump inhibitor**				< 0.001			< 0.001
Yes	16.8	4.2	95.8		13.4	86.6	
No	83.6	2.4	97.6		4.9	95.1	
**Education**				< 0.001			< 0.001
Beyond high school	58.6	2.0	98.0		4.7	95.3	
High school or below	41.4	3.6	96.4		8.6	91.4	
**Gender**				0.0330			< 0.001
Male	54.5	2.4	97.6		4.5	95.5	
Female	45.5	3.0	97.0		8.4	91.6	
**Age**				0.4950			0.2355
20–29	25.9	2.7	97.3		5.8	94.2	
30–39	22.7	2.3	97.7		5.7	94.3	
40–49	19.9	2.9	97.1		6.5	93.5	
50–59	15.8	3.2	96.8		7.2	92.8	
≥ 60	15.8	2.5	97.5		6.8	93.2	
**Race**				< 0.001			< 0.001
Hispanic	6.8	5.1	94.9		9.1	90.9	
Non-Hispanic white	61.4	2.2	97.8		5.7	94.3	
Non-Hispanic black	12.0	3.0	97.0		8.3	91.7	
Other	19.8	3.4	96.6		5.8	94.2	
**Marital status**				< 0.001			< 0.001
Married	52.7	1.5	98.5		3.8	96.2	
Widowed or separated	14.9	4.9	95.1		12.5	87.5	
Never married	32.4	3.6	96.4		7.5	92.5	
**Number of people in household**				0.1450			< 0.001
Living alone	10.2	3.5	96.5		9.4	90.6	
2–4	68.1	2.5	97.5		5.6	94.4	
≥ 5	21.7	2.8	97.2		7.1	92.9	
**Poverty income ratio**				< 0.001			< 0.001
< 2	15.3	5.2	94.8		13.0	87.0	
2–4	34.6	3.5	96.5		7.7	92.3	
> 4	43.0	1.2	98.8		2.8	97.2	
Missing data	7.1	2.6	97.4		6.5	93.5	
**BMI**				0.0200			< 0.001
Underweight	1.7	1.4	98.6		6.2	93.8	
Normal or healthy weight	30.4	2.5	97.5		5.6	94.4	
Overweight	34.1	2.4	97.6		5.4	94.6	
Obese	33.8	3.3	96.7		7.9	92.1	
**Smoking**				< 0.001			< 0.001
Current smoker	22.5	4.6	95.4		12.0	88.0	
Previous smoker	20.9	1.7	98.3		5.0	95.0	
Never smoker	56.6	2.3	97.7		4.5	95.5	
**History of cancer**				0.5334			< 0.001
Yes	8.8	28.3	71.7		9.6	90.4	
No	91.2	0.2	99.8		6.1	93.9	
**Number of comorbidities**^b^				< 0.001			< 0.001
0	95.6	2.6	97.4		5.8	94.2	
1	2.5	4.4	95.6		17.7	82.3	
2	1.0	7.7	92.3		18.2	81.8	
> 3	0.9	6.1	93.9		17.8	82.2	
**Diabetes**				< 0.001			< 0.001
Yes	4.3	5.8	94.2		19.2	80.8	
No	95.7	2.6	97.4		5.7	94.3	

^a^Rao–Scott χ^2^ statistics.

^b^Comorbidities included angina, coronary heart disease, heart failure, myocardial infarction and stroke.

In total, 2.7% and 6.3% of the weighted sample had suicidal ideation and depression, respectively, in the past 2 weeks (Table 1). About 16% of the weighted sample had been taking PPIs for more than 30 days. For the PPI users, 4.2% had suicidal ideation and 13.4% were depressed. Whereas the non-PPI users, only 2.4% and 4.9% had suicidal ideation and depression, respectively. Thus, the rate ratio (RR) for suicidal ideation was 1.75 (*P* < 0.001) and for depression was 2.73 (*P* < 0.001), indicating that the PPI users had 1.75 and 2.73 times the rate of suicidal ideation and depression compared to the non-PPI users.

**Figure 2:**
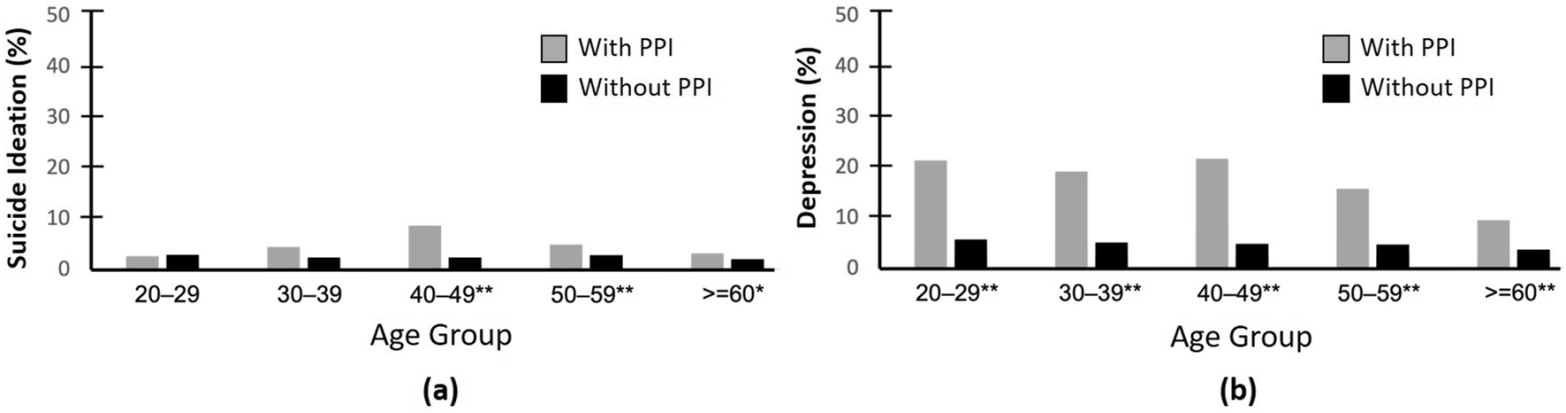
Weighted percentage of participants who had (**a**) suicidal ideation and (**b**) depression by age group for those taking or not taking PPIs. *P < 0.05; **P < 0.001.

The associations between PPI use and the two outcomes (suicidal ideation and depression) were further analysed by stratifying under different age groups (Fig. 2a). The PPI and non-PPI users had similar suicidal ideation rates in the 20–29-year age group but a higher prevalence of suicidal ideation was observed for the PPI users in all other age groups. The greatest difference was observed in the middle-aged group (40–49 years). With regard to depression, all the age groups had a significantly higher depression rate for the PPI users (Fig. 2b).

**Figure 3:**
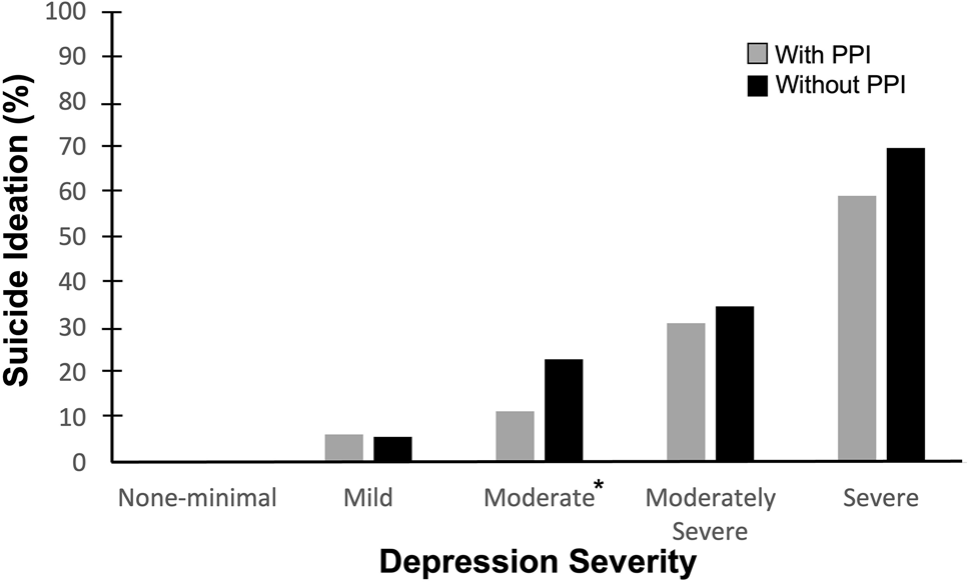
Weighted percentage of participants who had suicidal ideation by depression group for those taking or not taking PPIs. *P < 0.05. The suicidal ideation percentages of the none-minimal group for those taking or not taking PPIs were 0.27 and 0.33, respectively.

The participants were stratified into different depression severity groups to further investigate the association between PPI use and suicidal ideation. The depression severity groups were none-minimal, mild, moderate, moderately severe and severe depression. The results illustrated that the percentage of suicide ideation increased with the level of depression severity (Fig. 3). The PPI and non-PPI users had similar suicide ideation prevalence in the none-minimal and mild groups but with lower suicide ideation rates for the PPI users in all other groups (Fig. 3).

The bivariate Rao-Scott χ^2^ test assessed the associations between suicidal ideation and the covariates and found that most of them were significantly associated (*P* < 0.05) (Table 1). Participants with a low educational level, low PIR or a higher number of comorbidities were more likely to have suicidal ideation. Also, participants who were Hispanic, female, widowed or separated, obese, a current smoker or diabetic had a higher chance of having suicidal ideation. The RR between the high (> 4) and low (< 2) PIR was 4.3, which is the highest significant RR among all the other variables, followed by that of the widowed/separated and married participants (RR = 3.27). A higher percentage of participants with suicidal ideation also included those with a history of cancer or living alone, however these were not statistically significant (*P* > 0.05). There was no association between age and suicidal ideation (*P* > 0.05). With regard to depression, all the variables (except age) were significantly associated with the prevalence of depression (Table 1).

Three logistic regression models of suicidal ideation are provided in Table 2. The unadjusted model gave an OR of 1.79 and 95% CI of 1.35–2.37. This model revealed that the PPI users had 1.79 greater odds of having suicidal ideation than the non-PPI users. Among the three models in Table 2, the education, gender, race and age-adjusted model achieved the highest association (OR: 2.34; 95% CI 1.66–3.31). Similar to suicidal ideation, the PPI users were also more likely to have depression than the non-PPI users. Table 3 shows three logistic regression models of depression. The OR of the unadjusted model was 3.01 (95% CI 2.51–3.60); this association was slightly weaker than the other two adjusted models. The education, gender, race and age-adjusted model achieved the highest association (OR: 3.77; 95% CI 3.01–4.73).

**Table 2:** Weighted and adjusted odds ratios of PPI use and covariates with suicide ideation in 16,881 participants, NHANES 2005–2018.

Variable	Odds ratios (95% confidence intervals) of suicide ideation		
	Unadjusted	Adjusted model 1	Adjusted model 2
PPI (yes vs. no)	1.79 (1.35–2.37)	1.90 (1.36–2.66)	2.34 (1.66–3.31)
**Education**			
Beyond high school		Reference	Reference
High school or below		0.72 (0.57–0.91)	0.61 (0.48–0.77)
**Gender**			
Male		Reference	Reference
Female		1.25 (1.01–1.55)	1.21 (1.00–1.48)
**Race**			
Hispanic		Reference	Reference
Non-Hispanic white		0.37 (0.27–0.50)	0.41 (0.30–0.55)
Non-Hispanic black		0.50 (0.37–0.69)	0.58 (0.43–0.78)
Other		0.66 (0.48–0.91)	0.67 (0.48–0.92)
**BMI**			
Underweight		Reference	
Normal or healthy weight		2.08 (0.92–4.68)	
Overweight		2.06 (0.91–4.68)	
Obese		2.60 (1.15–5.75)	
**Smoking**			
Current smoker		Reference	
Previous smoker		0.46 (0.35–0.60)	
Never smoker		0.36 (0.23–0.49)	
**Number of comorbidities**^a^			
0		0.42 (0.24–0.74)	
1		Reference	
2		0.50 (0.24–1.06)	
> 3		0.68 (0.29–1.31)	
**Age**			
20–29			Reference
30–39			1.65 (1.15–2.36)
40–49			1.37 (0.89–2.10)
50–59			1.65 (1.06–2.56)
≥ 60			1.68 (1.10–2.56)

^a^Comorbidities included angina, coronary heart disease, heart failure, myocardial infarction and stroke.

**Table 3:** Weighted and adjusted odds ratios of PPI use and covariates with depression in 16,881 participants, NHANES 2005–2018.

Variable	Odds ratios (95% confidence intervals) of depression		
	Unadjusted	Adjusted model 1	Adjusted model 2
PPI (yes vs. no)	3.01 (2.51–3.60)	3.77 (3.01–4.73)	3.41 (2.67–4.34)
**Education**			
Beyond high school		Reference	Reference
High school or below		0.54 (0.46–0.63)	0.69 (0.59–0.81)
**Gender**			
Male		Reference	Reference
Female		1.86 (1.53–2.26)	2.05 (1.68–2.50)
**Race**			
Hispanic		Reference	Reference
Non-Hispanic white		1.56 (1.24–1.98)	0.48 (0.38–0.61)
Non-Hispanic black		0.89 (0.73–1.11)	0.75 (0.59–0.94)
Other		1.40 (1.16–1.70)	0.62 (0.49–0.78)
**Age**			
20–29		1.62 (1.24–2.21)	1.60 (1.21–2.21)
30–39		1.49 (1.14–1.96)	1.47 (1.10–1.96)
40–49		1.54 (1.17–2.01)	1.47 (1.11–1.96)
50–59		Reference	Reference
**Smoking**			
Current smoker			Reference
Previous smoker			0.33 (0.27–0.42)
Never smoker			0.32 (0.26–0.39)
**Diabetes**			
Yes			Reference
No			2.28 (1.68–3.10)

## Discussion

PPI use was found to be associated with depression and suicidal ideation in this cross-sectional study using the NHANES data of 16,881 participants between 2005 and 2018. The association between PPI use and depression was stronger than that between PPI use and suicidal ideation. All the eleven covariates were associated with depression but only six of them were associated with suicidal ideation (Table 1). They were the education, race, marital status, PIR, smoking and number of comorbidities.

Literature searches were performed on major scientific databases but no related information about the relationship between PPI use and suicidal ideation could be found. Thus, we believe that this is the first study to reveal the association between suicidal ideation and PPI use in a US national dataset. This study also further consolidated the association between PPIs and depression that was found in a previous study conducted with an Italian population-based dataset^12^. The pharmacological mechanisms of the psychological side effects of PPIs are not clear. One study suggested that PPIs may affect the microenvironment of the human nervous system by increasing the amount of Aβ-tau protein complex^40^. This may limit the beneficial effect of tau protein on the microtubule assembly and stabilization in the central nervous system, producing neurological and psychological adverse effects. Another study suggested that the psychological side effects of PPIs were potentially caused by the reduced absorption of nutrients, especially vitamin B_12_^4^. Vitamin B_12_ helps to maintain the health of the neurological system and a deficiency may cause cognitive impairment and neurological disorders^13^.

Another possible mechanism underlying the associations between PPI use and depression or suicidal ideation is PPI-induced hypomagnesemia. PPI may alter gastric acidity and reduce the absorption of minerals, such as magnesium^41^. Long-term use of PPI may cause hypomagnesemia. Magnesium is a necessary component in over 300 enzymatic reactions in humans^42^. A deficiency of magnesium may cause neurological, cardiovascular and musculoskeletal disorders^42^. A meta-analysis study found that an individual with hypomagnesemia has an overall increased 1.3-fold risk of depression^43^.

In this study, the selection criteria for PPI users with depression and suicidal ideation were those who had been taking PPIs for at least 30 days and 14 days, respectively. The reason was to ensure that the PPIs had enough exposure time to produce effects on the participants. However, the potential psychological effects of PPIs may gradually increase during long-term uses^44^. These effects may take months or even longer time to develop^44^. For example, the gradual change of gut microbiota can be changed gradually by taking PPIs^45^. A study indicated that PPIs favoured the growth of certain microorganisms by inhibiting their competitors^45^. This may have changed the gut microbiota and affected the absorption of micronutrients^46^, such as the psychotropics: vitamin B_12_ and magnesium. Thus, PPIs may take much longer than 30 days to produce the potential depression and suicidal ideation effects, and the PPI exposure time for some participants in this study was insufficient.

The prevalences of suicidal ideation and depression found in this study are similar to those reported in previous US population-based studies^32,47^, which were around 3% for suicidal ideation and 7% for depression. As indicated by the OR values of the logistic models in Tables 2 and 3, both suicidal ideation and depression were associated with PPI use. The association between depression and PPIs was higher than that of suicidal ideation. We believe that this is a reasonable outcome because suicidal ideation may be considered a symptom of severe depression^48^.

The selected logistic model between PPI use and depression (OR: 3.77; 95% CI 3.01–4.73) found in this US national population study agrees with that found in an Italian population-based study (OR: 2.38; 95% CI 1.02–5.58)^12^. The sample size and sample selection criteria were different, for instance, this study involved participants between 20 and 85 years old, whereas the Italian study involved only those aged 75 years and over. Nevertheless, both studies suggested that the PPI users had more than twice the odds of having depression than the non-PPI users.

Figure 2b demonstrates the percentage difference of depressed participants between the PPI and non-PPI users among different age groups. The oldest group (≥ 60 years) obtained the smallest difference between the PPI and non-PPI users. This may explain why the OR between PPIs and depression obtained in the Italian study was lower than that found in this study. The same circumstances were also observed for suicidal ideation (Fig. 2a), which may be due to the amount of gastric acid output declining with ageing^49^. PPI can decrease gastric acidity and reduce the absorption of psychotropics (such as vitamin B_12_ and magnesium). Thus, the difference in gastric acidity between PPI users and non-users may vary with age. The elderly may have smaller differences in acidity between the PPI users and non-users than those at a younger age. This may lead to the participants in the older groups being more prone to depression^50^, causing the lower percentage differences of depressed participants observed between the PPI users and non-users in the older age groups. Further studies are required to investigate this matter.

Another result worth discussing is that the 40–49-year age group exhibited the greatest difference in suicidal ideation rate between the PPI and non-PPI users (Fig. 2a), whereas the difference in depression rate between the PPI and non-PPI users for the same age group was similar to that for the other age groups (Fig. 2b). As suicidal ideation may be considered as a symptom of severe depression, this result indicated that PPIs may have produced stronger adverse psychological effects in the middle-aged group. Again, further studies are required to investigate this matter.

Figure 3 demonstrates the associations between PPI usage and suicidal ideation under different severity of depression. The suicidal ideation prevalences were lower in the PPI users at moderate to severe depression stages. A possible explanation for this interesting finding is the potential drug–drug interactions between PPIs and antidepressants. The participants at moderate to severe depression stages in this study may have taken antidepressants which are known to be associated with a reduced risk of suicide^51^. A clinical study with 46,352 participants indicated that the co-administration of PPIs and the common antidepressants (citalopram, escitalopram and sertraline) can increase the serum concentration of the antidepressants up to 93.9%^52^. This indicates PPIs may be able to potentiate the suicidal prevention effects of antidepressants. Again, further studies are required to investigate this matter.

The bivariate Rao-Scott χ^2^ test indicated that most of the covariates were associated with suicidal ideation or depression (Table 1). This agrees well with the study performed by Diep et al., which was aimed at finding the association between cannabis use and suicidal ideation using similar covariates and the same dataset (NHANES 2005–2018) as this study^32^.

In this study, the cancer covariate was significantly associated with depression but not with suicidal ideation. This seems to be in contrast with previous studies, which suggested that cancer patients were more likely to have suicidal thoughts^53,54^. This contrast may be caused by the different sampling criteria and interview questions. In this study, the participants were being asked whether or not they had suicidal thoughts in the past 2 weeks. However, the interview question to assess cancer information was: ‘*Have you ever been told that you had cancer or a malignancy?*’. Thus, the participants in this study included those who had recovered from cancer more than 2 weeks ago and therefore did not have cancer when asked about their suicidal ideation. This is different from the other studies, in which the participants had cancer when their suicidal thoughts were assessed.

There is literature showing that people who were living alone were more prone to have depression^55,56^ and suicidal ideation^57^. However, in this study, the participants who lived alone or with five or more people were likely to have depression or suicidal ideation. These associations were statistically significant for depression but not for suicidal ideation.

This study has several limitations that may affect the interpretation of the results. Suicidal ideation may be considered as sensitive information and there is a chance that the participants may not have responded accurately to the NHANES questionnaires. All the NHANES data were cross-sectional, with no control variables for accurate outcome measurements, meaning that a causal relationship between the variables and outcomes cannot be determined. Thus, this study cannot determine whether PPI use affected suicidal ideation or vice versa. Other potential cross-sectional study limitations include selection bias, information bias, recall bias and detection bias^58^.

Another limitation of this study is the lack of information on the dosages and formulations of the PPIs. One study has demonstrated that the pharmacological effects of PPIs are dose-dependent^59^. Thus, if the dosage information was made available in the NHANES dataset, the association between the dosage of PPIs and suicidal ideation or depression could be found.

In this study, the BMI covariate was found to be associated with both suicidal ideation and depression. However, studies have shown that PPIs may cause weight gain^60^, therefore the BMI may not be truly independent of the exposure variable in this study.

The exclusion of other covariates found in the literature is also a limitation of this study. Examples include a history of psychiatric disorder^61^, experience of suicidal behaviour in relatives or friends, previous suicidal attempts, psychological behaviour such as hostility and inferiority, and genetic information^62^. The pharmacological effects of PPIs can be affected by the genetic polymorphism of the participants, thus PPIs may affect people differently according to their genes^63^. This is particularly significant for the effects on cognitive performance^10^. The inclusion of the above-mentioned data as covariates in this study could further reinforce our regression models; however, the NHANES 2005–2018 dataset did not include this information.

This study investigated the effects of six different PPIs as a group and did not consider the effect of each of them alone. This was because the sample size of each PPI was not large enough for adequate statistical power. Future study may consider the individual effect of different PPIs. Further investigations may also be required to specify the extent of the results from this study, such as the dose-dependent relationship between PPIs and suicidal ideation, and the association between patient’s genetic polymorphisms and the severity of depression and suicidal ideation.

## Conclusion

This study revealed that the recent use of PPIs was associated with suicidal ideation and depression among US non-institutionalized adults using data from a nationally representative survey. The associations found in this study proposed the potential psychological effects of PPI use. Further prospective longitudinal studies are required to investigate the magnitude of these associations and explore their implications, such as the possibility of mood monitoring requirements during PPI use.

## Data Availability

All data used for analysis are freely available via online public domains (https://www.cdc.gov/nchs/nhanes/index.htm).
